# Salt in Milk for Children

**Published:** 1889-01

**Authors:** 


Salt in Milk for Children.—Dr. A. Jacobi (*Arch. of Ped.*) says that the addition of sodium chloride prevents the solid coagulation of milk by either rennet or gastric juice. The cow's milk ought never to be given without table salt, and the latter ought to be added to woman's milk when it behaves like cow's milk in regard to solid curdling and consequent indigestibility. Habitual constipation of children is influenced beneficially, since not only is the food made more digestible, but the alimentary secretions, both serous and glandular, are made more effective by its presence.

